# Association between Illness Perception and Emotional Status in Iranian Patients after Heart Transplantation

**Published:** 2020-01

**Authors:** Nazila Shahmansouri, Mehrdad Salehi, Ali Reza Bakhshandeh, Roya Sattarzadeh Badkoubeh, Masoumeh Lotfi-Tokaldani, Ahmad Ali Noorbala, Azadeh Mashayekhi

**Affiliations:** 1 Psychosomatic Medicine Research Center, Imam Khomeini Hospital Complex, Tehran University of Medical Sciences, Tehran, Iran.; 2 Imam Khomeini Hospital Complex, Tehran University of Medical Sciences, Tehran, Iran.; 3 Tehran Heart Center, Tehran University of Medical Sciences, Tehran, Iran.

**Keywords:** *Perception*, *Depression*, *Anxiety*, *Heart transplantation*

## Abstract

**Background:** Heart transplantation is a major procedure which imposes high emotional stress on patients. Illness perception (IP) is a psychological issue which affects psychological adjustment after transplantation. This study aimed to investigate the association between IP and emotional status in Iranian post-heart transplantation patients.

**Methods:** The present cross-sectional study, conducted between 2018 and 2019 in Imam Khomeini Hospital, Tehran, Iran, recruited 121 post-heart transplantation patients. IP was measured using the Brief Illness Perception Questionnaire (B-IPQ), and emotional status was measured using the Hospital Anxiety and Depression Scale. The association between IP and depression/anxiety was assessed.

**Results:** Men comprised 80.2% of the study population. The mean age of the participants was 43.9±12.95 years. Definite caseness for depression and anxiety was reported in 11.6% and 18.2% of the participants, respectively. The median score of IP was 55. The association between anxiety and IP in total IP and the 3 dimensions of IP was statically significant (P=0.015, P=0.018, P=0.002, and P=0.023 for the cognition, emotion, and understanding dimensions and the total IPQ, respectively). Additionally, the association between depression and IP was significant (P=0.001, P=0.029, and P=0.002 for the cognition and emotion dimensions and the total IPQ, correspondingly, except for the understanding dimension). Furthermore, lower levels of anxiety in the patients showed a greater impact on IP than did depression.

**Conclusion:** There was a significant association between IP and depression and anxiety in our study population. Therefore, the diagnosis and management of anxiety and depression in heart transplantation patients may improve IP. The cross-sectional design of the present study precluded an investigation of the causality between IP and emotional status.

## Introduction

Heart transplantation is a lifesaving intervention in patients with heart failure and some other cardiac diseases.^1^^, ^^2^ The first successful heart transplantation in Iran was performed by Dr. Mandegar in Shariati Hospital, affiliated to Tehran University of Medical Sciences, in July 1993.^3^ In a single-center registry report, the 1-month and 1-year survival rates after heart transplant were 82.6% and 70%, respectively.^4^

Post-heart transplantation psychological distress may, directly and indirectly, affect physiological health.^5^ Lesko and Hawkin^6^ stated that surgical procedures such as heart transplantation in humans led to psychological stress. The prevalence of emotional distress among heart transplantation patients has been studied by some other researchers as well. Brocks et al^7^ showed that 10% and 11% of their 477 cardiac transplantation patients had moderate or severe depressive symptoms, respectively, and 14% and 12% had mild or high levels of anxiety, correspondingly.

 A phenomenological study on life with a transplanted heart in the Iranian population indicated that the first year after transplantation, especially in first 2 to 3 months, was a critical time and that the heart recipients could not tolerate and handle all the pressure and stress.^8^

Deshields et al.^9^ indicated that psychological distress (i.e., depression and anxiety), along with several indices of cognitive function, improved after transplantation in their study population. They also suggested that anxiety and depression were positively correlated with the number of organ rejection episodes.

There are also numerous studies on how emotional distress can affect other aspects of psychological well-being in heart transplantation patients. By way of example, Doering et al.^10^ suggested that anxiety and depressive symptoms mediated the effects of perceived control on the quality of life in heart transplantation patients. Additionally, scholars who have explored psychological responses to health threats have conceptualized “illness perception”, which affects the way patients cope with the disease (e.g., by problem-solving activities or emotional responses to illness).^11^^, ^^12^ Illness perception was originally defined by Leventhal et al.^11^ as the patient’s representation of his or her health condition and the way that the patient conceptualizes health threat as an illness. In tandem with illness perception is the emotional representation of the illness, which is presumed to result in coping behaviors and appraisal processes.^11^

Given the impact of psychological factors on patients’ quality of life, treatment adherence, and survival rates, it is of great importance to study the relationship between illness perception and emotional status among cardiac transplantation patients.

## Methods

The present cross-sectional study examined 121 patients who had undergone cardiac transplantation surgery since 2001 in Imam Khomeini Hospital, Tehran, Iran. The proposal of the study was approved by the Ethics Committee of Imam Khomeini Hospital Complex, Tehran University of Medical Sciences (Approval ID:IR.TUMS.IKHC.REC.1397.009). 

The inclusion criteria were comprised of age over 18 years, lack of intellectual disability and cognitive problems, and the provision of informed consent. Patients failing to meet these conditions were excluded from the study.

Sociodemographic information such as age, sex, the educational level, and marital status were collected through a questionnaire, and some clinical data such as age at transplantation, the time elapsed from transplantation, the cardiac disease type (congenital or non-congenital), the number of cardiac rehospitalizations after transplantation, and cigarette and substance use after transplantation were collected from the patients’ clinical records.

The patients completed the Brief Illness Perception Questionnaire (B-IPQ) and the Hospital Anxiety and Depression Scale (HADS) questionnaire during medical follow-up visits. Informed consent was obtained from all the participants.

The B-IPQ is a 9-item questionnaire, the first 8 questions of which are Likert-type items with 0 to 10 points. They assess consequence, timeline, personal control, treatment control, identity, illness concern, illness understanding, and emotional response to the illness. Item 9 is the causal item in which patients are asked to list 3 of the most important causes of their illness.^13^ The B-IPQ identifies 3 dimensions of illness perception. The summation of the scores of Question 1 to Question 5 indicates the cognitive dimension, the summation of the scores of Question 6 to Question 8 represents the emotional dimension, and the score of Question 7 denotes the understanding dimension of illness perception. The Farsi version of the B-IPQ was validated by Bazzazian et al., who reported that the questionnaire had good internal consistency, construct validity, concurrent validity, and cross-cultural validity (Bazzazian S, Besharat MA. Reliability and validity of a Farsi version of the brief illness perception questionnaire. Procedia Soc Behav Sci 2010;5:962–965.).

In addition, the HADS questionnaire was used to assess emotional distress in the participants. HADS is a 14-item scale that generates ordinal data. Seven of the items relate to anxiety, and 7 relate to depression. Each item is scored between 0 and 3, which means that the total score can range between 0 and 21 for either anxiety or depression. Scores 0 to 7 denote the absence of anxiety or depressive symptoms, scores 8 to 10 are interpreted as the likelihood of anxiety or depression, and scores 11 to 21 show definite caseness for anxiety or depression.^14^^, ^^15^ The Farsi version of the questionnaire was validated by Montazeri et al.,^16^ who reported a Cronbach’s alpha coefficient (to test reliability) of 0.78 for the HADS anxiety subscale and 0.86 for the HADS depression subscale. Zigmond and Snaith^14^ created this scale specifically to avoid reliance on the aspects of these conditions that were also common in physical illness, such as fatigue and insomnia, in order that it could be used to examine the anxiety and depression associated with physical illness.

The continuous variables are described as the mean with the standard deviation (SD) or the median with the interquartile range (IQR_25-75%_) boundaries for the variables with normal and skewed distributions, respectively. The normality of the variables was assessed using histogram charts as well as the abovementioned descriptive measures. The categorical variables were expressed as the frequency and the percentage. Linear regression models were conducted to evaluate the unadjusted and adjusted associations between the B-IPQ score and its 3 dimensions (as dependent variables) and the emotional depression and anxiety status variables (as independent variables). All the statistical analyses were conducted using IBM SPSS Statistics for Windows, version 23.0 (Armonk, NY: IBM Corp.). 

## Results

The data on 121 participants, comprised of 80.2% male and 19.8% female patients, were analyzed. The mean age of the study population was 43.9±12.95 years. The mean age at transplantation was 39.55±13.19 years. The median (IQR_25-75%_) time elapsed from transplantation among the patients was 36 (14–78) months. The median number of cardiac hospitalizations after heart transplantation was 1 (0–3), and the median time in the waiting list for heart transplantation was 3 (1–6) months. Furthermore, 5.8% of the respondents had been diagnosed with congenital cardiac diseases before heart transplantation. Table 1 represents the study population’s sociodemographic and clinical profile.

**Table 1:** Sociodemographic profile of the participants*

Marital Status	
Single	23 (19)
Married	92 (76)
Widow	5 (4.1)
Divorced	1 (0.8)
Sex	
Male	97 (80.2)
Female	24 (19.8)
Education	
Illiterate	7 (5.8)
Primary school	22 (18.2)
Below diploma	22 (18.2)
Diploma	47 (38.8)
Bachelor’s degree and above	23 (19)
Cardiac Disease Type	
Congenital	7 (5.8)
Non-congenital	114 (94.2)
Cigrette Smoking after Transplantation	
Yes	4 (3.3)
No	117 (96.7)
Substance Use after Transplantation	
Yes	3 (2.5)
No	118 (97.5)

* Data are presented as numbers (%).

In the B-IPQ, the median scores (25th–75th percentile) for the 3 dimensions of cognition, emotion, and understanding, as well as the total score, were 36 (28–40), 17 (10–20), 0 (0–1), and 55 (36–60), respectively. Higher scores indicate a more threatening view of the illness.

According to the HADS scoring, 11.6% and 18.2% of the participants were definite cases of depression and anxiety, respectively, 20.7% and 22.3% were probable cases of depression and anxiety, correspondingly, and 67.8% and 59.5% demonstrated no anxiety or depressive symptoms, correspondingly.

Table 2 and Table 3 present the unadjusted and adjusted effects of depression and anxiety on the total B-IPQ scores and the 3 dimensions of illness perception. Adjustments were also made for the following variables: sex, age at transplantation, the post-transplantation time, the waiting time for transplantation, the number of cardiac rehospitalizations after transplantation, and cigarette and substance abuse after transplantation. The scores of anxiety and the total B-IPQ, as well as the scores of all 3 dimensions of illness perception, were significantly correlated before and after the adjustments. However, the dimensions of depression and understanding showed no significant association after and before the adjustments, whereas the association between depression and the total B-IPQ, cognition, and emotion was statistically significant.

After the adjustments for confounding factors, a 1-score increase in probable depression versus no depression resulted in an average of a 7.29-score increase in the total B-IPQ score and a 1-score increase in definite depression versus no depression resulted in an average of a 13.56-score increase in the total B-IPQ score.

Moreover, after the adjustments, a 1-score increase in probable anxiety versus no anxiety resulted in an 8.83-score increase in the total B-IPQ score and a 1-score increase in definite anxiety versus no anxiety resulted in an 8.69-score increase in the total B-IPQ score.

As is presented in Table 3, after the adjustments, there was no significant difference between probable and definite anxiety regarding their effects on illness perception. In other words, even low levels of anxiety had higher effects on illness perception than did depression.

**Table 2:** Unadjusted and adjusted effects of depression on the total B-IPQ and the 3 dimensions of illness percep

	Unadjusted		Adjusted	
	B* (95% CI)	P	B* (95% CI)	P
Probable vs. no depression				
Cognition	4.62 (0.35-8.89)	0.034	3.89 (-0.33-8.12)	0.071
Emotion	3.23 (0.37-6.09)	0.027	2.89 (0.00-5.78)	0.050
Understanding	0.47 (-0.59- 1.53)	0.384	0.50 (-0.56-1.56)	0.354
Total B-IPQ	8.32 (1.64-15.01)	0.015	7.29 (0.58-13.99)	0.033
Definite vs. no depression				
Cognition	8.88 (3.48- 14.28)	0.001	8.74 (3.35-14.13)	0.001
Emotion	4.52 (0.90- 8.14)	0.014	4.11 (0.43-7.80)	0.029
Understanding	0.73 (-0.60- 2.08)	0.283	0.69 (-0.66-2.05)	0.315
Total B-IPQ	14.14 (5.68-22.60)	0.001	13.56 (5.01-22.10)	0.002

B-IPQ, Brief illness perception questionnaire

* Regression coefficient

**Table 3:** Unadjusted and adjusted effects of anxiety on the total B-IPQ and the 3 dimensions of illness perception

	Unadjusted		Adjusted	
	B* (95% CI)	P	B* (95% CI)	P
Probable vs. no anxiety				
Cognition	5.31 (1.00- 9.61)	0.016	5.23 (1.02-9.45)	0.221
Emotion	3.35 (0.53- 6.16)	0.020	3.40 (0.59-6.20)	0.017
Understanding	0.28 (-0.73- 1.30)	0.581	0.19 (-0.80-1.20)	0.701
Total B-IPQ	8.94 (2.28-15.61)	0.008	8.83 (2.23-15.44)	0.009
Definite vs. no anxiety				
Cognition	4.33 (-0.31- 8.97)	0.068	2.99 (-1.79-7.79)	0.015
Emotion	4.23 (1.19- 7.27)	0.006	3.85 (0.65-7.04)	0.018
Understanding	1.68 (.58- 2.78)	0.003	01.84 (0.70-2.98)	0.002
Total B-IPQ	10.24 (3.05- 17.44)	0.005	8.69 (1.17-16.21)	0.023

B-IPQ, Brief illness perception questionnaire

* Regression coefficient

## Discussion

In the current study, we demonstrated a significant association between anxiety and the 3 dimensions of illness perception, as well as an association between depression and illness perception, except for the understating dimension. Furthermore, a low level of anxiety in our patients exerted greater effects on illness perception than did depression. Thus, managing anxiety in heart transplantation patients can improve illness perception. 

As is presented in the Results, the score of the understanding dimension of illness perception indicated that the patients had a good understanding of their illness. This is in line with the results of a study by Janelle et al.,^17^ who demonstrated that heart transplantation patients appeared to have a good understanding of their health condition. The median, minimum, and maximum scores of understanding dimension in their study were 2, 1.2, and 2.6, respectively.

A small number of studies have addressed the association between illness perception and emotional status among heart transplantation patients. Shahmansouri et al.^18^ studied illness perception in a sex-based analysis among patients with premature coronary artery disease 8 years after diagnosis. They suggested that their female subjects with mixed anxiety/depression scored significantly lower than did their treatment control group. These respondents were also far more concerned about their illness and scored lower than did their male counterparts in the understanding dimension. Nonetheless, as regards anxiety only or depression only, Shahmansouri and colleagues found no significant difference between their female and male patients. Contrary to these results, our study revealed significant associations between both depression and anxiety and illness perception. 

Morgan et al.^19^ found that illness perception played a significant role in psychological issues (e.g., depression and anxiety) among their heart failure patients. They suggested that a significant proportion of variance in the depression and anxiety scores could be explained by illness perception. This finding chimes in with the results of the current study insofar as we found that the association between illness perception and depression and anxiety was significant after adjustments for confounding factors.

Kugler et al.^20^ in an investigation into emotional adjustment before and after heart transplantation showed that depression and state-trait anxiety levels were higher in the case group during the transplantation waiting time than those among healthy subjects in the control group. However, 20 days after transplantation, the depression and state-trait anxiety levels in the patients were not significantly different from those of the healthy control group. In contrast, we found that post-transplantation depression and anxiety levels were not significantly different from those of the general population,^21^ which is concordant with the findings by Kugler and coworkers.

Brock et al.^7^ reported that in 477 cardiac transplantation patients, 10% and 11% had, respectively, mild and severe depression and 14% and 12% had, correspondingly, mild and severe anxiety. In our study, the prevalence rates of moderate and severe anxiety and mild depression are higher than those in the study by Brock and colleagues; nonetheless, the prevalence of severe depression in our study is similar to that in their investigation. Many social, economic, and cultural factors in our population can justify the discrepancies in the findings between our study and that by Bruck et al.

We assessed emotional status only after heart transplantation; therefore, we were unable to make any before-after comparisons. We recommend that the correlation between psychological tests and mental state examinations be explored in further studies. Finally, the cross-sectional design of the present study precluded the determination of any causality between illness perception and emotional status. Future studies with larger sample sizes and without the aforementioned limitations are suggested.

## Conclusion

The current study showed a significant association both between anxiety and all the aspects of illness perception and between depression and illness perception except for the understanding dimension. Furthermore, a low level of anxiety in our patients had greater effects on illness perception than did depression. Accordingly, the diagnosis and management of anxiety and depression in this population can improve illness perception.

## Notes:

***This paper should be cited as: ***Shahmansouri N, Salehi M, Bakhshandeh AR, Sattarzadeh Badkoubeh R, Lotfi-Tokaldani M, Noorbala AA, Mashayekhi A. Association between Illness Perception and Emotional Status in Iranian Patients after Heart Transplantation. J Teh Univ Heart Ctr 2020;15(1):12-17.
